# Mechano-Transduction: From Molecules to Tissues

**DOI:** 10.1371/journal.pbio.1001996

**Published:** 2014-11-18

**Authors:** Beth L. Pruitt, Alexander R. Dunn, William I. Weis, W. James Nelson

**Affiliations:** 1 Department of Mechanical Engineering, Stanford University, Stanford, California, United States of America; 2 Department of Chemical Engineering, Stanford University, Stanford, California, United States of America; 3 Cardiovascular Institute, Stanford University, Stanford, California, United States of America; 4 Department of Structural Biology, Stanford University, Stanford, California, United States of America; 5 Department of Molecular and Cellular Physiology, Stanford University, Stanford, California, United States of America; 6 Department of Biology, Stanford University, Stanford, California, United States of America

## Abstract

External forces play complex roles in cell organization, fate, and homeostasis. Changes in these forces, or how cells respond to them, can result in abnormal embryonic development and diseases in adults. How cells sense and respond to these mechanical stimuli requires an understanding of the biophysical principles that underlie changes in protein conformation and result in alterations in the organization and function of cells and tissues. Here, we discuss mechano-transduction as it applies to protein conformation, cellular organization, and multi-cell (tissue) function.

## Abstract 2

Biological mechano-transduction and force-dependent changes scale from protein conformation (â„« to nm) to cell organization and multi-cell function (mm to cm) to affect cell organization, fate, and homeostasis.

## Introduction

All the cells and tissues of the body are subject to external forces. These may include fluid shear stress (as in the vasculature), osmotic forces (in the urinary tract), mechanical load (in bone and muscle), and stretch (in the lung and intestine), as well as the stiffness of the extracellular matrix (ECM) that surrounds most cells. These external forces can affect the shape and intracellular organization of cells, their proliferation and migration, and their intercellular interactions; they influence the development of embryos as well as cell function and homeostasis in the adult. Moreover, many disease states—including cardiac hypertrophy, arthrosclerosis, and cancer—are characterized by abnormal changes in these forces or loss of the normal cellular response to them [1],[2]. Understanding the nature of these external forces and how cells sense and respond appropriately to them is a complicated problem that ranges in scale from protein conformation (Å–nm) to cell organization (nm–µm) and multi-cell function (µm–mm–cm).

## Biophysical Principles

Mechano-transduction can be defined as a cellular process that converts a mechanical input, for example, stretching or fluid flow, into intracellular signal transduction. The detailed molecular mechanisms that underlie mechano-transduction can be complex. However, a few basic physical principles are sufficient to understand much of how mechano-transduction is thought to occur.

**Figure 1:**
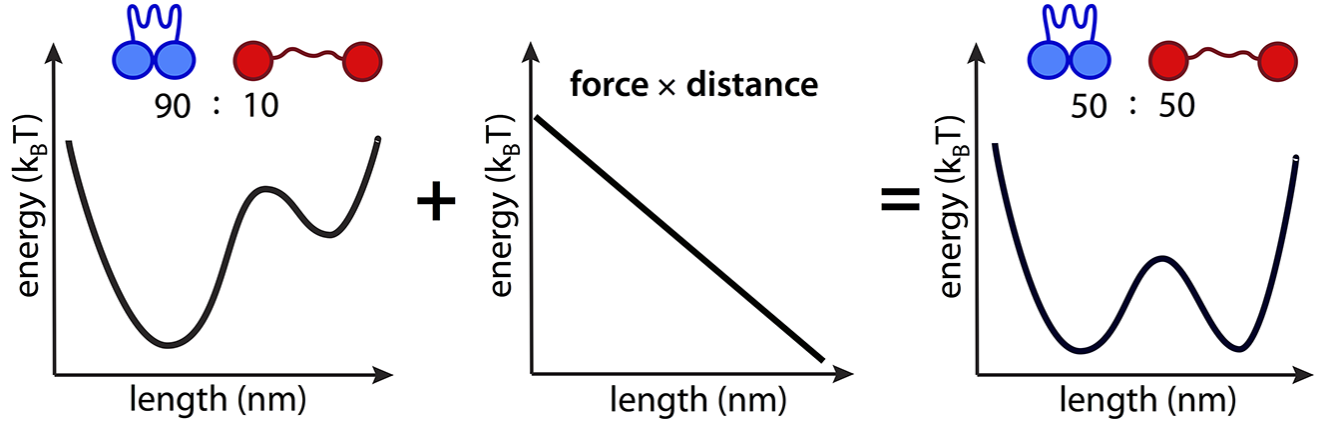
Mechanical force effects protein conformational equilibrium. Proteins exist in a conformational equilibrium in which different states are populated according to their relative energies. Mechanical force shifts the equilibrium among pre-existing states. Consider a protein in equilibrium between compact and extended conformations *A* (blue) and *B (*red*)*. The corresponding equilibrium constant *K*  =  [*B*]/[*A*] relates to the free energy difference *ΔG* between states as: *K*  =  exp(-*ΔG*/*k_B_T*), where *k_B_* is Boltzmann's constant and *T* is absolute temperature. An applied load *F* stabilizes the extended conformation by a mechanical work term of *F* times distance, *δ*, leading to a new equilibrium constant *K′*  =  exp[(-*ΔG*+*Fδ*/*k_B_T*] [4]. In effect, force shifts the energetic balance between the compact and extended states, increasing the amount of protein in the extended conformation by a factor of five in the illustration above. It is useful to remember that since *k_B_T* has units of energy, it can be expressed in units of force multiplied by distance and is 4.2 pN nm at physiological temperature. Thus, pN forces acting over nm distances are sufficient to meaningfully shift the equilibrium between conformations.

In well-studied examples of mechano-transduction, specific proteins undergo force-induced alterations in conformation that are coupled to changes in catalytic activity or affinity for binding partners (Figure 1). In this way, mechanical load triggers biochemical changes that can propagate via canonical signal transduction pathways (see sections on Protein and Cell Levels). In order to understand more precisely how proteins “feel” force, it is useful to recall that mechanical work, a form of energy, can be expressed either as joules or as newton meters (force × distance). Intuitively, moving an object against a resisting force over some distance requires an input of energy. The same holds true at the molecular level: stretching a protein two nanometers (nm; a typical protein dimension) against a load of two piconewtons (pN; the force generated by a single myosin motor protein) requires an input of 4 zeptojoules (zJ; 4×10^−21^ joules).

A zeptojoule is an unimaginably small amount of energy, but it can be related to physically intuitive quantities. The amount of work necessary to stretch one mole of proteins (6.02×10^23^ molecules) 2 nm against 2 pN of force, as above, is 4×10^−21^ J×6.02×10^23^, or 2.4 kJ per mole. Is this a lot or a little? As it turns out, this energy is approximately 20 times less than the energy derived from ATP hydrolysis under physiological conditions. In fact, it is comparable in magnitude to the thermally generated jostling that proteins experience under ambient conditions (see Figure 1).

To understand how cells overcome this challenge, we can take advantage of the equivalence of mechanical work and chemical free energy at the molecular level. Proteins exist in a conformational equilibrium in which different states are populated according to their relative energies. Mechanical force can shift the equilibrium between these pre-existing states (Figure 1). Importantly, the equilibrium between two states depends exponentially on applied force. In the example above, 2 pN acting over 2 nm will shift the conformational equilibrium by a factor of 2.6. Higher forces shift the equilibrium dramatically: 10 pN (approximately 5 myosin molecules) acting over 2 pN shifts the equilibrium by a factor of 130. In summary, even very small forces generated by single motor proteins can alter a conformational equilibrium enough to potentially modulate downstream signaling. Modestly larger forces can dramatically alter the sensor protein conformation, and hence signal transduction.

In most cases, mechanical load accelerates the dissociation of protein-ligand bonds. However, a variety of cellular adhesion proteins show *catch bond* behavior [1], in which mechanical load produces changes in protein conformation that lead to a higher affinity for a binding partner (see sections on Proteins and Cells) [3],[4]. In addition, several myosin motors (for example, myosin I, II, V, and VI) have higher affinity for adenosine diphosphate in the presence of mechanical load. This increases the fraction of their catalytic cycle spent bound to actin, thus causing them to transition from molecular cargo transporters to cytoskeletal anchors as load increases [5]–[11]. The fact that load can either destabilize or stabilize protein-ligand interactions depending on the specific protein/ligand pair offers rich possibilities for the regulation of downstream signaling by mechanical load (see sections on Protein and Cell Levels).

The description of mechano-transduction above technically applies only when the system is at pseudo-equilibrium. On sufficiently short timescales (<msec), protein conformational changes will fail to keep up with a rapidly changing external force, leading to the failure of equilibrium-based models. Interestingly, these timescales are characteristic of mechano-transduction in muscle contraction, hearing and touch. Theoretical advances have provided a firm basis for relating how proteins respond to rapidly changing external forces to more readily measured equilibrium thermodynamic properties, for example, in the context of mechanical unfolding of RNA and proteins [12]–[14]. Aside from a few examples, theoretical approaches from non-equilibrium statistical mechanics have not yet been applied to the study of cellular mechano-transduction [15]. Theoretical tools developed in the context of single-molecule biophysics may prove useful to mechanobiologists, particularly those who study physiological systems with fast dynamics such as the examples listed above.

## Protein Level

Understanding how fundamental physical principles are encoded in protein conformation is central to understanding how force is converted into biochemical signals [1],[3]–[11]. At first glance, the relationship between the protein conformations we visualize by structural methods, such as crystallography, and the force-dependent functional changes we see in cells, is not obvious. However, if force is viewed as an allosteric modulator that shifts the equilibrium between pre-existing functional states, then the structures trapped in crystals may reflect conformations that are populated significantly in vivo only under mechanical load. Moreover, when force is coupled to increases in the affinity of a protein for its ligand (e.g., in catch bonds), the presence of a saturating concentration of ligand can shift the equilibrium to reveal force-dependent conformations—as has been demonstrated by structural analyses of the bacterial adhesin FimH that forms the tip of the bacterial adhesion structure called the fimbria [16], as well as the mammalian adhesion proteins selectins [17] and integrins [18],[19].

Empirical approaches have been used to test the idea that a given crystal structure might represent one conformation on a force-dependent pathway to activation. For example, a particular conformation can be covalently “locked” by introducing disulfide bonds into the protein and then tested to see whether force-dependent changes in binding affinity are inhibited (this approach has been used with fibronectin [20]). Mutations can also be introduced into a protein to disrupt interfaces thought to be involved in the force-induced pathway to activation (as has been used with selectins [21]–[23]). Alternatively, protein conformation can be examined by electron microscopy or solution scattering methods in the presence of antibodies that promote force-dependent ligand binding (as has been done with integrins [24]). These data support the notion that crystal structures can reveal force-dependent conformations.

Structural studies have revealed that force-sensitive proteins have multiple domains and flexible interdomain interfaces that allow the protein to pass through (or “sample”) multiple conformations. For example, a bent or hook-shaped conformation can open up to a straighter arrangement of domains along the direction of applied force; this allows distal regions of the protein to act as a lever arm to transmit the force needed to open the interface and promote the needed conformational changes. Such structural changes have been observed in FimH [25], as well as in selectins and integrins [24],[26]. In these cases, force responsiveness can be “tuned” by adjusting the length and flexibility of the lever arm. The resulting interfacial changes are transmitted to distal portions of the domain and affect partner binding.

Force-induced conformational states can be coupled in many ways. For example, tension on the bacterial adhesin FimH changes the interface between its N-terminal lectin domain and its pilin domain. This causes an elongation and narrowing of the lectin domain β-barrel that stabilizes a high-affinity mannose-binding site at the opposite end of the domain [16],[25]. In a similar way, force-induced changes in L-selectin remodel the sugar-binding site: the interaction between the N-terminal lectin and the EGF-like domain is coupled to changes in loops in the lectin domain that connect elements of secondary structure [23],[26]. Force applied to the ligand-binding domain of integrin β-chains (so-called “headpiece opening”) also transforms a relatively bent two-domain structure to one that is straight [24]. This change results in a 75 Å movement of the more rigid domains of the β-chain that are parallel to the cell surface, thus creating a wide separation from the α subunit.

**Figure 2:**
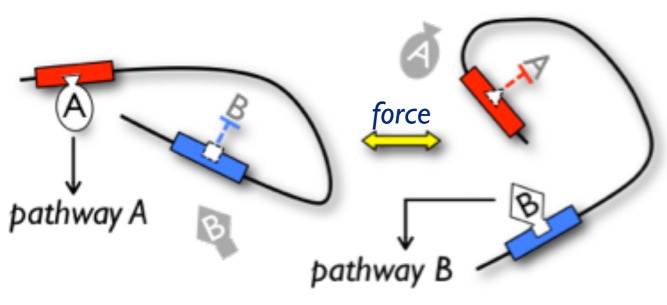
Regulation of protein binding and function by conformational changes. Left: This protein conformation allows protein A to bind and activate its pathway A but blocks binding of protein B, so pathway B is inactive. Right: A change in protein conformation induced by an external force, for example, inhibits the binding of protein A and allows protein B to bind, and so activate, pathway B.

In addition to the conformational remodeling examples above, force-induced partial unfolding of a protein can expose otherwise cryptic binding sites for a partner (Figure 2). A well-studied example is the focal adhesion protein talin (see also the following section), in which the N-terminal FERM domain binds to the cytoplasmic tail of the integrin β-subunit, and the C-terminal five-helix bundle domain binds to actin [27]. These two domains are connected by a large rod domain comprising a series of helical bundles that, in the absence of force, is relatively compact but flexible [28]–[30]. Force applied by actomyosin contraction causes the helical bundles in the rod domain to unfold, exposing binding sites for vinculin. The binding of vinculin to these sites then stabilizes a repacked structure in which one of the talin helices forms a five-helix bundle with four helices of vinculin [28],[30],[31]. Thus, force shifts the conformational equilibrium to favor the “exposed” vinculin-binding helix. Other examples of cryptic binding sites exposed by application of force have been observed in von Willebrand factor and fibronectin [32],[33].

The stability of protein domains that undergo the types of rearrangement described above must be such that the conformational equilibrium changes significantly only at an appropriate force threshold, but also must be sufficient to avoid denaturation at the highest relevant forces. Thus, protein domains that can undergo change are likely to be tuned to a restricted range of physiological forces. The effect of force on protein conformation might also extend to higher-order assemblies. Scaffolding proteins such as talin recruit kinases, phosphatases, GTP exchange factor (GEF)s, and GTPase activating protein (GAP)s to adhesion sites. Mechanical tension might alter the interactions of these bound signaling molecules: for example, force applied to a scaffolding protein that binds both a kinase and its substrate might enable or disable phosphorylation of the target protein.

## Cell Level

**Figure 3:**
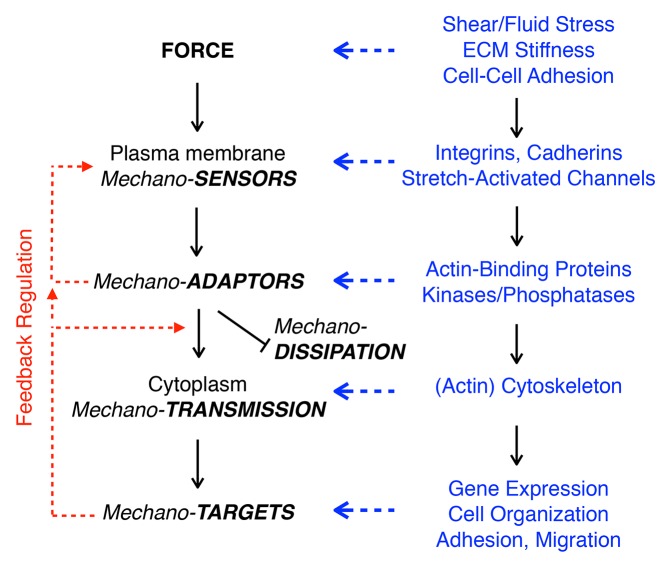
Pathways linking force at the cell surface to intracellular signaling and downstream effectors. External force is detected by mechano-sensors in the plasma membrane, which link to intracellular adaptors that transmit mechanical signals to targets in the cell. Right: examples of processes and proteins involved. These pathways may exhibit feedback regulation (see main text for details).

Conformational changes in response to mechanical force occur in many large protein assemblies and subcellular structures, which can lead to local and global changes in cell organization, behavior, proliferation, and differentiation. Although various types of membrane organization such as caveolae [34],[35] and membrane bending by BAR domain–containing proteins [36] respond to mechanical forces at the cell surface, here we will focus on cell adhesion to the ECM. How cells sense and convert external forces into intracellular signals has been studied extensively in tissue culture in the context of single cell adhesion to the ECM (Figure 3) [1], and multicellular organization [37] (see the section on Multi-cell Level).

As we have seen above, a characteristic of many proteins that link external forces from the ECM to intracellular signaling is that they undergo unfolding in response to force or tension. Fibronectin is a major component of the ECM with multiple binding sites for other ECM proteins and integrins. Fibronectin undergoes changes in conformation (unfolding) upon stretching in vivo [38] and in vitro [39]. This results in exposure of binding sites that were buried in the folded domains, and inactivation of binding sites exposed on the surface of folded domains. As a consequence, stretching alters ECM organization and thus the binding of ECM components to integrins in the cell membrane. Integrins also undergo conformational changes upon binding extracellular ligands in the ECM, resulting in their conversion from a weak to a high-affinity binding conformation [40], consistent with the catch bond behavior [1] (see section on Biophysical Principles).

A large number of cytoplasmic proteins are either directly bound to integrins (e.g., talin), or locally recruited around integrins (e.g., vinculin, p130^cas^, zyxin, and filamin A) [41]. Together, these proteins assemble into a focal adhesion complex that is linked to the actomyosin cytoskeleton. Talin and p130^cas^ undergo tension-mediated unfolding resulting in exposure of additional binding sites, such as vinculin-binding sites in talin [42] (discussed in the section on Protein Level), or modification of binding sites by Src-mediated phosphorylation of p130^cas^ [43]. Studies using a Forster resonance energy transfer (FRET) tension sensor (TsMod) also showed that vinculin is under tension at focal adhesions [44]. As a result, ECM–integrin adhesion is reinforced by the recruitment and modification of proteins that cluster integrins and recruit the actomyosin cytoskeleton [45]. Only a subset of such complexes may be stabilized in response to force, however. Other actin-associated protein–protein interactions may be “slip bonds,” which unbind in response to force, leading to disassembly of protein complexes and local dissipation of forces.

Force-mediated reorganization of focal adhesion complexes results in changes in the regulation of Rho family small GTPases. For example, the Rac-GAP FilGAP is released in a tension-dependent manner from filamin A and may locally decrease Rac-controlled actin and membrane activity [46]. By contrast, the Rho-GEFs LARG and GEF-H1 are activated by force applied to integrins and may locally increase Rho-dependent actomyosin contractility [47].

Changes in the organization and contractility of the actin cytoskeleton provide short- and long-range transmission of force to downstream targets (Figure 3). Short-range transmission alters the organization of focal adhesions and the strength of cell binding to the ECM [1], whereas long-range transmission alters cell shape [48], fate, and function [49]. One important target is the nucleus and the regulation of gene expression [50]. All elements of the cytoskeleton are bound to a protein complex called the linker of nucleoskeleton and cytoskeleton (LINC) complex that bridges the nuclear envelope [51]. This complex mediates force transmission across the nuclear envelope, resulting in nuclear deformation [52] and alterations in chromatin structure and organization [53] that affect gene expression. A number of diseases of muscle tissue (muscular dystrophy and cardiomyopathies) result from mutations in LINC complex components [54]. This coupling between the nucleus and cytoskeleton is essential for cell migration, wound healing, cancer metastasis, and development [55], reviewed in [56].

How forces are integrated through cell–cell adhesions is less well understood [2], but progress is starting to be made [57]–[61] (see recent reviews [62],[63]). For example, recent studies have used the TsMod FRET sensor [44] to detect actomyosin-dependent tension on the cytoplasmic domain of the cell–cell adhesion protein E-cadherin in cultured cells [59], and in border cells during their migration in the *Drosophila* ovary [64]. Many of the underlying principles identified in cell–ECM binding may apply to cell–cell adhesion complexes. A recent study showed that alpha-catenin in the cadherin-catenin adhesion complex binds actin under force, and is a catch-bond [65].

## Multi-cell Level

Whereas the analysis of force-induced effects on cells in culture can be addressed by the use of imaging and biochemistry and can be manipulated by drugs or protein depletion, it has been difficult to use these approaches to analyze tissue homeostasis in vivo [66]. However, this is changing rapidly with advances in ways to grow multi-cell structures and tissues on specialized surfaces that mimic the stiffness of ECM in vivo, and the use of strain arrays to induce strain across tissues in vitro in a controlled manner.

Since the 1930s, the effects of external force on arrays of cells have been studied by implanting cells into contrived mechanical environments [67], by using feeder layers to create soft substrates [68], and by engineering methods to mechanically stimulate explanted cells (reviewed in [69]). Other studies have employed static technologies such as cell patterning (the growth of cells on defined shapes and surface areas), modulated substrate stiffness, or passive force measurements. The results have been correlated with functional outcomes such as cell migration and stem cell differentiation [49],[70]. Moreover, some organisms are amenable to direct perturbation and observation of tissue morphogenesis under modified loads (e.g., compression, laser dissection, and contact probes) [71]–[73]. A comprehensive understanding of force transfer across and within a tissue will require dynamic, integrated approaches using quantitative tools to apply and measure forces and deformations.

**Figure 4:**
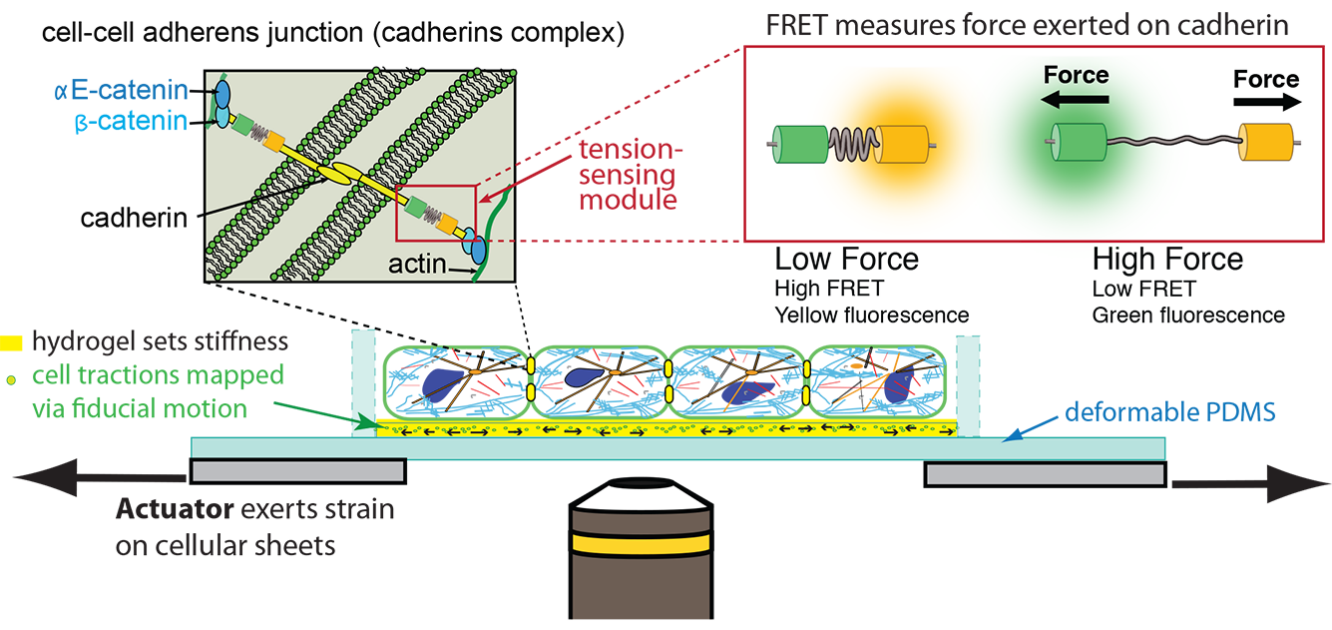
Analysis of force transduction at cell–cell junctions. In a monolayer of epithelial cells growing on a hydrogel of known stiffness, cell–cell adherens junctions mechanically link one cell to one another. These junctions comprise transmembrane cadherin proteins that bind to each other in the intracellular space and on the cytoplasmic face of the plasma membrane form ternary complexes with αE- and β-catenin; the molecular identity of the linker to the actin cytoskeleton and other proteins in the tension-sensing module remain unknown. Förster resonance energy transfer (FRET) probes engineered into key proteins allow us to measure quantitatively the molecular-scale forces the cells exert on each other in response to a macroscopic stretch applied by an actuator.

A major challenge is to understand how cells distribute their load between cell–ECM and cell–cell contacts (Figure 4). In the case of multi-cell structures, it is particularly difficult to infer the forces felt by any one cell. Mechanical forces during epithelial sheet migration [74] or folding of epithelial tubes [75], for example, have been observed at a tissue level but not correlated to subcellular pathways in the cells. Yet we know changes in loading or the mechanical environment do modulate numerous intracellular pathways involved in, for example, changes in the cytoskeleton or cell proliferation, and changes in cell function such as increased endothelial permeability and leukocyte transmigration [76].

Mechanical forces [77],[78] and protein patterning (reviewed in [79]) can determine the alignment of cells in multi-cellular arrays, which, in turn, can affect functions like muscle cell contractility [80] and Ca^++^ handling [81]. Interestingly, both of these organizing factors—mechanical alignment and protein patterning—elicit similar patterns of gene expression [82]. By contrast, dynamic or steady flow forces applied in parallel or perpendicular to the cell alignment activate distinct signaling programs [60]. These dual-stimuli experiments indicate that we are only just beginning to appreciate the likely importance of ECM–cell surface cues, tissue topology and strain history in development and disease. A combination of cell co-cultures with dual-stimuli mechanical environments might thus open new avenues for creating functional tissue models.

Microfabricated devices (miniature, fabricated structures of cm–mm–µm scales used to study cell and tissue structure and function under controlled conditions) are uniquely suited to apply forces in the physiological range of nN–µN at cell (nm–µm) and multi-cell (µm–mm) scales. Cell displacements can be measured and forces inferred by reference to a model of material properties (for example, the stiffness or Young's modulus of the elastic substrate material) [83]. For example, quantification of cell–substrate traction forces can be tracked by the displacement of beads suspended in polymer hydrogels [84]–[88] or by the deformation of micropillars made of elastomer, a viscoelastic polymer with low Young's modulus [89],[90]. Similar approaches have been used to calculate forces at cell–cell contacts and the interplay between forces at cell–ECM and cell–cell contacts [91]–[93]. Elastomer-based and hydrogel (a cross-linked hydrophilic polymer that is optically transparent and flexible)-based platforms have been particularly successful because their material properties can be well matched to the stiffness (in the range of Pa–MPa) and elastic strains (0.01%–100%) of cells or tissues [94]; however, these platforms would benefit from integration into more physiological dynamic assays.

Mechanically actuated devices are needed to provide relevant quantifiable measures of the effects of external force on cell growth, differentiation, and maturation. For example, cyclically stretched perforated membrane microfluidic devices (also called “organ-on-a-chip,” a 3-D microfluidic cell culture chip that simulates the activities, mechanics, and physiological response of an organ) have been used with endothelial and epithelial cells to mimic the human lung air–blood barrier [95]. These “lung-on-a-chip” studies found that stretch increased the inflammatory response of cells to nanoparticle exposure, and they highlight the importance of using physiologically relevant mechano-stimulation. When isolated epithelial explants from the developing salivary gland [96],[97], kidney [98], breast [99], intestine [100], or lacrimal gland [101] are embedded in 3-D gels of reconstituted basement membrane protein, they form branches in the presence of exogenously applied growth factors (reviewed in [102]–[104]). Likewise, in reconstituted mammary epithelium, mechanical stresses exerted by the epithelium initiate nascent branches and direct branching morphogenesis [105],[106]. Tissue culture cell lines can also be induced to differentiate into 3-D structures in the appropriate 3-D matrix environment (forming, for example, a “gut-on-a-chip” [107]). These approaches are being extended to build integrated “human-on-a-chip” models consisting of interconnected compartments, in which each compartment contains a different organ, derived from specific cell types, connected by a microfluidic circulatory system [108].

Further integration of such in vivo–like simulated systems may eventually bridge the gap between standard cell culture and animal studies. Collaboration between structural biologists, biophysicists, cell biologists, and mechanical engineers is going to be required to build these new systems and enable a broad understanding of how structural changes at the molecular level result in functional changes in tissues and whole organisms.
